# The Reproducibility and Relative Validity of a Food Frequency Questionnaire for Identifying Iron-Related Dietary Patterns in Pregnant Women

**DOI:** 10.3390/nu14112313

**Published:** 2022-05-31

**Authors:** Mayra Lizeth Navarro-Padilla, María Fernanda Bernal-Orozco, Joan Fernández-Ballart, Barbara Vizmanos, Norma Patricia Rodríguez-Rocha, Gabriela Macedo-Ojeda

**Affiliations:** 1 Health Sciences University Center, University of Guadalajara, Guadalajara 44340, Mexico; ln.mayra.navarro@gmail.com; 2 Institute of Translational Nutrigenetics and Nutrigenomics, Department of Clinics of Human and Health Reproduction, Growth and Child Development, Health Sciences University Center, University of Guadalajara, Guadalajara 44340, Mexico; fernanda.bernal@academicos.udg.mx (M.F.B.-O.); bvizmanos@yahoo.com.mx (B.V.); 3 Area of Preventive Medicine and Public Health, Faculty of Medicine and Health Sciences, Universitat Rovira i Virgili, IISPV, 43201 Reus, Spain; joan.fernandez-ballart@urv.cat; 4 CIBERobn (CB06/03) Instituto de Salud Carlos III, 28029 Madrid, Spain; 5 Department of Public Health, Regional Institute for Public Health Research, Health Sciences University Center, University of Guadalajara, Guadalajara 44340, Mexico; norma.rodriguez@academicos.udg.mx; 6 Department of Public Health, Institute of Research in Biomedical Sciences, Health Sciences University Center, University of Guadalajara, Guadalajara 44340, Mexico

**Keywords:** pregnancy, dietary iron intake, dietary patterns, food frequency questionnaire, reproducibility, relative validity

## Abstract

Analyzing pregnant women’s iron intake using dietary patterns would provide information that considers dietary relationships with other nutrients and their sources. The objective of this study was to evaluate the reproducibility and relative validity of a Qualitative Food Frequency Questionnaire to identify iron-related dietary patterns (FeP-FFQ) among Mexican pregnant women. A convenience sample of pregnant women (*n* = 110) completed two FeP-FFQ (FeP-FFQ1 and FeP-FFQ2) and a 3-day diet record (3DDR). Foods appearing in the 3DDR were classified into the same food groupings as the FeP-FFQ, and most consumed foods were identified. Exploratory factor analysis was used to determine dietary patterns. Scores were compared (FeP-FFQ for reproducibility and FeP-FFQ1 vs. 3DDR for validity) through intraclass correlation coefficients (ICC), cross-classification, Bland–Altman analysis, and weighed Cohen kappa (κw), using dietary patterns scores tertiles. Two dietary patterns were identified: “healthy” and “processed foods and dairy”. ICCs (*p* < 0.01) for “healthy” pattern and “processed foods and dairy” pattern were 0.76 for and 0.71 for reproducibility, and 0.36 and 0.37 for validity, respectively. Cross-classification and Bland–Altman analysis showed good agreement for reproducibility and validity; κw values showed moderate agreement for reproducibility and low agreement for validity. In conclusion, the FeP-FFQ showed good indicators of reproducibility and validity to identify dietary patterns related to iron intake among pregnant women.

## 1. Introduction

Anemia is a health problem that affects over 1.5 billion people worldwide, and its prevalence is higher among >5-year-old children and pregnant women [1]. Thus, it is considered one of the leading global public health challenges. In Mexico, the health of women of reproductive age is affected by conditions such as high blood pressure (6.8% in women aged 20–39 years), hypercholesterolemia (18.6% in women aged 20–39 years), overweight (26.9% in adolescents; 36.3% in women aged 20–49 years) and obesity (14.1% in adolescents, 38.5% in women aged 20–49 years). The prevalence of anemia among women between 12–49 years of age is 17.5% for non-pregnant women while it is almost twice as high for pregnant women (34.9%) [2]. During pregnancy, anemia is associated with intrauterine growth restriction, low birthweight [3], perinatal death, and maternal mortality from hemorrhage [4].

According to the World Health Organization (WHO), anemia during pregnancy is defined as <11.0 g/dL hemoglobin concentrations during the first and third trimesters and <10.5 g/dL during the second trimester [5,6]. Among pregnant women, the world prevalence of anemia is 40.1% [7], and it can be as higher as 56% among low and middle-income countries [8], with iron deficiency being the most common cause.

During pregnancy, iron is essential because it is a crucial component of hemoglobin for oxygen supply, and it is necessary for diverse enzymatic reactions [9]. Thus, requirements and intestinal absorption are increased due to the expansion of red blood cell numbers and the transfer of higher iron quantities to the placental structures and fetus, especially during the second and third trimesters of pregnancy [4].

Iron intake is an important modifiable factor affecting iron status during pregnancy [10]. Pregnant women’s iron levels depend on different aspects: the quantity consumed through foods and supplements, the gestational trimester, body iron stores (requirement of at least 500 mg [11]), and iron-type (heme and non-heme). However, the consumed iron absorption process is the factor that most affects body iron bioavailability and homeostasis [12,13].

Non-heme iron absorption is affected by promoters like vitamin C, vitamin A and peptides containing cysteine and fiber, and inhibitors such as tannins, phytates, polyphenols, phosphorus, and calcium [14]. In contrast, heme iron is inhibited only in the presence of calcium [12].

Because nutrients are not consumed separately but in combination, it is crucial to consider pregnant women’s diet in its entirety when dietary factors associated with iron status are investigated [15]. For this purpose, analyzing dietary patterns is highly useful because these consider the whole diet, how the foods are consumed, and their different combinations. Therefore, their use might overcome the limitations of studying foods and nutrients individually and their associations between dietary intake and iron status [16].

An initial nutriments screening of pregnant women is needed to assess the prevalence of anemia during pregnancy. The Food Frequency Questionnaire (FFQ) is the most used tool to assess diet because it provides long-term diet intake data [15,17]. Numerous studies have used FFQs to establish diverse macro-and micro-nutrient dietary patterns. However, very few have reported the validity and reproducibility of FFQ to determine iron-related dietary patterns [18].

Therefore, this study aimed to design and validate a FFQ to identify iron-related dietary patterns among pregnant women.

## 2. Materials and Methods

### 2.1. Study Participants

The research was conducted in Guadalajara, Mexico, from March to September 2018, including pregnant women between 20 and 40 years of age, in either their second or third trimester of pregnancy and attending outpatient service at the Maternal and Child Hospital “Esperanza López Mateos”, a public hospital providing attention to pregnant adolescents and low-income women without social security. Exclusion criteria were women with multiple pregnancies, chronic disease diagnostic, or pregnancy complications (specifically, preeclampsia and gestational diabetes; gastrointestinal disorders were nor considered as exclusion criteria).

A convenience sampling with consecutive cases was used. The recommended sample size for the validity of semiquantitative food frequency questionnaires is 110 participants, approximately, considering a power of 80% and α = 0.5 [19]. Based on this, 140 participants were included at the beginning of the study, considering possible follow-up losses.

### 2.2. Study Design

The participants completed the qualitative Food Frequency Questionnaire through an interview to identify iron-related dietary patterns (FeP-FFQ) at baseline (FeP-FFQ1), which could be at either their 2nd or 3rd gestational trimester. During this first interview, sociodemographic and anthropometric data were collected, and participants were trained on how to complete a three-day diet record (3DDR), with two weekdays and one weekend day. The training was provided during the first interview and consisted of explaining how to fill out the diaries with five steps: the time and place of the meal, name of the dish, dish ingredients, type of ingredient (e.g., whole milk, light milk), amount consumed. These steps were also written on the diet record form along with an entry example. Participants were also instructed on standard cooking measures (tablespoon, teaspoon, cups). At the end of the training, participants were asked to write down one entry example to verify the instructions were clear and clarify questions. Participants were also instructed to take pictures of their reported meals and drinks whenever possible. They were asked to submit their 3DDR at their next interview and were contacted by phone in advance to verify they had no questions. One month later, the participants submitted their 3DDR and it was verified that they had been completed correctly; at this point in time they also completed the second FeP-FFQ (FeP-FFQ2).

### 2.3. FeP-FFQ Development and Application

The FeP-FFQ is an adaptation of Beck et al.’s [18] qualitative Fe-FFQ. Foods rich in iron, calcium, vitamin C, vitamin A, and fiber were added, considering those of frequent consumption in Mexico. Following Zhou’s [20] suggestion, foods with at least 3% of the daily iron recommended intake for Mexican pregnant women were included (SAME [21]). The qualitative FeP-FFQ includes a total of 76 items divided into ten categories: vegetables; fruits; grains and starchy foods; legumes; meat, poultry, cured meats and eggs; fish and seafood; dairy; snacks, sweets and desserts; alcoholic beverages; and other beverages. Participants were asked how often they ate each food during pregnancy, with nine answer options: never or rarely, 1–3 times/month, once a week, 2–4 times/week, 5–6 times/week, once a day, 2–3 times/day, 4–6 times/day, and ≥7 times/day.

### 2.4. Measurements

Procedures for data collection are presented below.

#### 2.4.1. Sociodemographic, Anthropometric Data and Iron Status

Sociodemographic (e.g., age, sex, civil status, education, socioeconomic status) and anthropometric (e.g., pregestational weight, current weight, height) data were collected at the moment participants were recruited, when they could be at either the 2nd or 3rd trimester of pregnancy. Data were collected through a questionnaire designed for this study and applied by a trained nutritionist. Socioeconomic status was evaluated and classified using the AMAI (Mexican Association of Market Intelligence and Opinion Agencies) [22] scores. Weight was measured using a Tanita scale, model TBF-300A with a 0.1 kg precision, and height was measured with a Seca portable stadiometer, model 213. Age was calculated from the participants’ birthdates. Current and pregestational body mass indexes (BMI) were calculated by dividing weight in kilograms by height in meters squared. Current BMI was classified using the Atalah curves for the Latin-American population [23]. Hemoglobin status data were obtained from the medical records at the 3^rd^ trimester of pregnancy.

#### 2.4.2. Diet Records

During the first interview, women received the 3DDR form, were instructed on how to fill it correctly, and were asked to submit it four weeks later, during their second appointment. Each participant filled two diet records of weekdays and one of a weekend day, specifying mealtime, place, and time of consumption. They were asked to write down the name of the dish and each ingredient and the type of food (e.g., whole milk, skim milk, lactose-free milk); and when possible, to take photos of all meals and drinks consumed during the three reported days. During the second appointment, 3DDRs were collected and screened for missing information. Subsequently, all foods in the records were counted, added up, and categorized manually according to the 76 items in the FeP-FFQ. For analysis purposes, when participants reported consuming two different food items of the same group at the same mealtime (e.g., strawberries and orange), it was registered as a two-time consumption of the food group, except for water, which was registered as one intake for every 250 mL consumed during a day. For dishes where the participants provided recipes, ingredients were assigned individually to the corresponding FeP-FFQ item. Some of the foods reported in the 3DDR did not match any of the FeP-FFQ items, which were excluded from analyses.

### 2.5. Statistical Analysis

Descriptive analytics were performed for quantitative variables and are presented as means and standard deviations (SD), while qualitative variables are presented as frequencies and percentages.

To evaluate the reproducibility and relative validity of each FeP-FFQ item, the intake frequency was adjusted for each participant and each food consumed by using the midpoint as the level of consumption. Then values were converted to weekly intake frequency (e.g., 1–3 times/month = 2 times/month = 0.5 times/week). These weekly values were later converted to a 3-day consumption frequency to align with the 3DDR (e.g., 0.5 times/week divided by seven and multiplied by 3 = 0.21 times in 3 days). Additionally, all food items reported in the 3DDRs were categorized into the 76 items of the FeP-FFQ.

#### 2.5.1. Identification of the Most Frequently Consumed Food Items

To identify dietary patterns, first, the most frequently consumed food items were identified by obtaining the means and standard deviations of the consumption frequency of each food item from the 3DDR, FeP-FFQ1, and FeP-FFQ2. Food items not identified as frequent consumption in the 3DDR and both FeP-FFQ were excluded from the factor analyses. Thirty-two out of the 76 items emerged as of higher frequency of consumption.

#### 2.5.2. Identification of Dietary Patterns from the FeP-FFQs and the Diet Records

Spearman correlation was performed to measure the reproducibility of the most frequently consumed food items from the questionnaire (FeP-FFQ1 and FeP-FFQ2), while for relative validity, Pearson’s linear correlation was used for the consumption frequency of each item in the FeP-FFQ1 and 3DDR. Paired t-tests were used to compare food items’ intake frequency mean differences: FeP-FFQ1 vs. FeP-FFQ2 for reproducibility; and FeP-FFQ1 vs. 3DDR for relative validity, with a significant *p*-value of <0.05. The effect size was obtained for the significant differences between the questionnaires using the formula: effect size r = √t2/(t2 + d*f*) (where t = t statistic produced by paired *t*-test and d*f* = degrees of freedom). An effect size of 0.1 indicates a small effect, 0.3 a medium effect, and ≥0.5 a large effect [22].

Subsequently, a factor analysis with varimax rotation was used for FeP-FFQ1, FeP-FFQ2, and 3DDR to identify dietary patterns. The Kaiser–Meyer–Olkin measure of sampling adequacy and Bartlett’s test *p* values were used to determine the presence of relationships between variables in the factor analysis, with values of >0.5 and <0.05, respectively.

The relative validity and reproducibility of dietary patterns derived from FeP-FFQ1 were examined by calculating Pearson linear correlation coefficients, ICC, and cross-classification between diet pattern scores obtained from FeP-FFQ1 and the 3DDR (relative validity), and FeP-FFQ1 and FeP-FFQ2 (reproducibility).

Finally, diet pattern scores were divided into tertiles and reproducibility (FeP-FFQ1 vs. FeP-FFQ2) and validity (FeP-FFQ vs. 3DDR) analyses were complemented with the Bland–Altman method and weighted Kappa Cohen coefficient to assess the level of agreement between methods based on Landis and Koch [24] classification, where κw values between 0.21 and 0.40 indicate fair agreement, between 0.41 and 0.60 moderate agreement, between 0.61 and 0.80 good agreement, and >0.81 very good agreement.

All statistical analyses were performed using IBM-SPSS software version 21.

## 3. Results

### 3.1. Participant Characteristics

A total of 140 women were initially invited to participate and completed the first FeP-FFQ; 30 were excluded because they did not complete all the required questionnaires; therefore, 110 participants completed the study. Participant characteristics are detailed in Table 1. Mean age was 26.89 years (SD 5.58). The mean monthly family income was 409.82 USD (SD 315.52), and from this, 199.42 USD (SD 77.84) is used as a monthly food stipend. Most of the participants live in free union (54.5%), have an education level of secondary school (46.4%), are housewives (80.9%), have a family composed of a mother, father, and children (73.6%), and are low-middle class (31.8%). The mean pregestational BMI was 26.0 kg/m^2^ (SD 5.7), and the majority of participants were classified as “normal weight” (43.6%). In contrast, the mean current BMI was 28.8 kg/m^2^ (SD 5.6) and “overweight” was the most common classification among participants (35.5%).

### 3.2. Food Intake Frequency

From the three questionnaires (FeP-FFQ1, FeP-FFQ2 y 3DDR), 32 food items with the most frequent consumption were obtained (Table 2). Mean consumption fluctuated between 15.54 (for water) to 0.90 (for sausages and ham) times/3 days. Slight differences in frequency of consumption were found for the 32 items between FeP-FFQ2 and 3DDR compared to FeP-FFQ1. On the other hand, the mean intake frequency reported was higher in both FeP-FFQs than in the 3DDR for the 32 food items except water and beef. For most items, consumption frequency was significantly different between FeP-FFQ1 and 3DDR. This contrasts with the comparison between both FeP-FFQs, where significant differences were only found for 10 out of 32 items. Through paired t-tests, it was found that the consumption frequency was overestimated for FeP-FFQ1 in 19 out of 32 items (59.4% of the foods) compared to the 3DDR (*p* < 0.01): corn tortilla, tomato, citrus fruits, lime, stone fruits, homemade beans, banana and fried plantain, apple, lettuce, carrot, melon, watermelon, sour cream, rice, yogurt (drinking yogurt included), pasta soup, papaya, ice-cream/sorbet/popsicles, and beef.

### 3.3. Identification of Dietary Patterns

The Kaiser–Meyer–Olkin measure of sampling adequacy was 0.761 for FeP-FFQ1, 0.640 for FeP-FFQ2, and 0.491 for 3DDR (>0.5 acceptable), and Bartlett’s test *p* values were all 0.001 (<0.001 acceptable). Identified dietary patterns were: (a) “healthy” dietary patterns consisting of chilies, lime, onion, banana, apple, citrus fruits, melon, stone fruits, carrot and papaya; and (b) “industrialized food and dairy” pattern including soda, ice-cream, rice, pasta soup, sausages, and cheese (Table 3). The dietary patterns obtained from the FeP-FFQ1, FeP-FFQ2, and 3DDR explained 31.81%, 27.84%, and 17.24% of the variance in the food intake scores for each dietary pattern, respectively.

### 3.4. Reproducibility and Validity of Dietary Patterns

Pearson correlation coefficients for the reproducibility of the dietary pattern scores from the factorial analysis between FeP-FFQ1 and FeP-FFQ2 suggested good reproducibility: 0.60 for the “healthy” pattern and 0.55 for the “industrialized food and dairy” pattern (*p* < 0.01); while the ICC were 0.76 and 0.71 (<0.01), respectively. Validity correlations of the dietary pattern scores between FeP-FFQ1 and 3DDR were: 0.11 for the “healthy” pattern (*p* > 0.05) and 0.20 for the “industrialized food and dairy” pattern (*p* < 0.05). ICC for the validity of the dietary pattern scores were 0.36 for the “healthy” pattern and 0.37 for the “industrialized food and dairy” pattern (*p* < 0.01). Cross-classification of the reproducibility of dietary pattern scores found 58.2% of participants classified in the same third for “healthy” and 51.9% for “industrialized food and dairy” pattern. A low percentage was classified in the opposite third for the “healthy” pattern (5.5%) and 11.9% for the “industrialized food and dairy” pattern.

Moreover, a fair and significant agreement for the validity of both patterns was found (κw = 0.12 for ”healthy” and κw = 0.14 for “industrialized food and dairy”); and a moderate and significant agreement for reproducibility of the “healthy” pattern (κw = 0.47) and “industrialized food and dairy” pattern (κw = 0.32).

**Figure 1:**
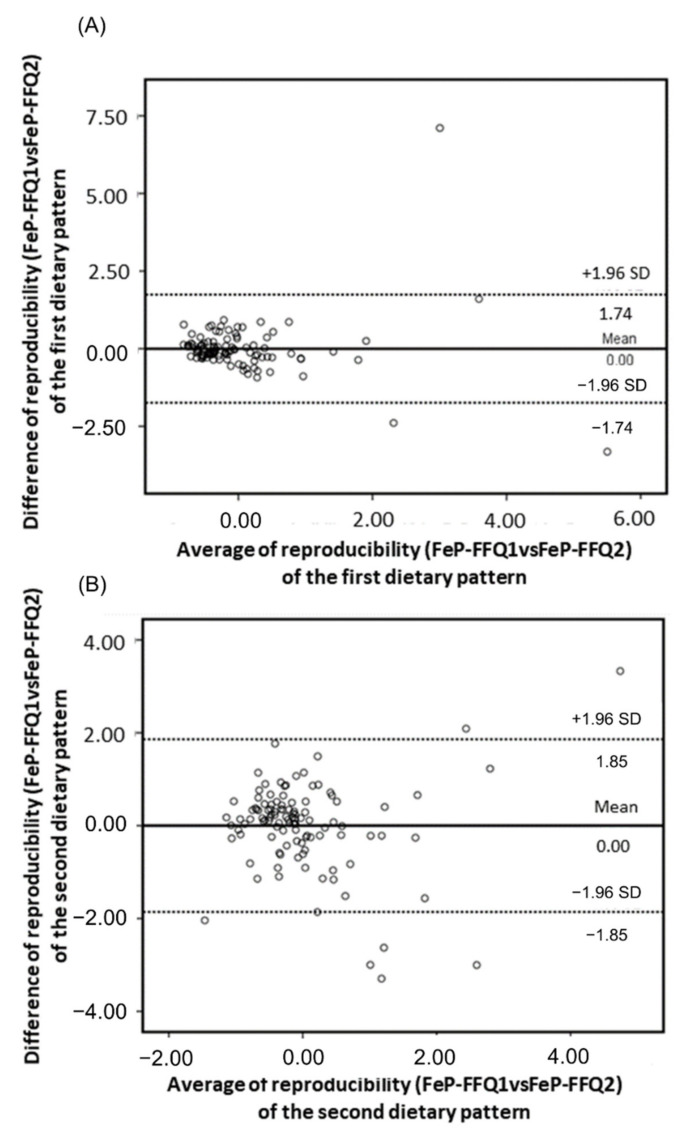
Bland–Altman plots for dietary patterns reproducibility between FeP-FFQ1 and FeP-FFQ2: (**A**) “healthy” pattern agreement, (**B**) “industrialized food and dairy” pattern agreement. The solid line represents the mean difference, and the dotted lines represent the agreement limits (SD ±1.96).

**Figure 2:**
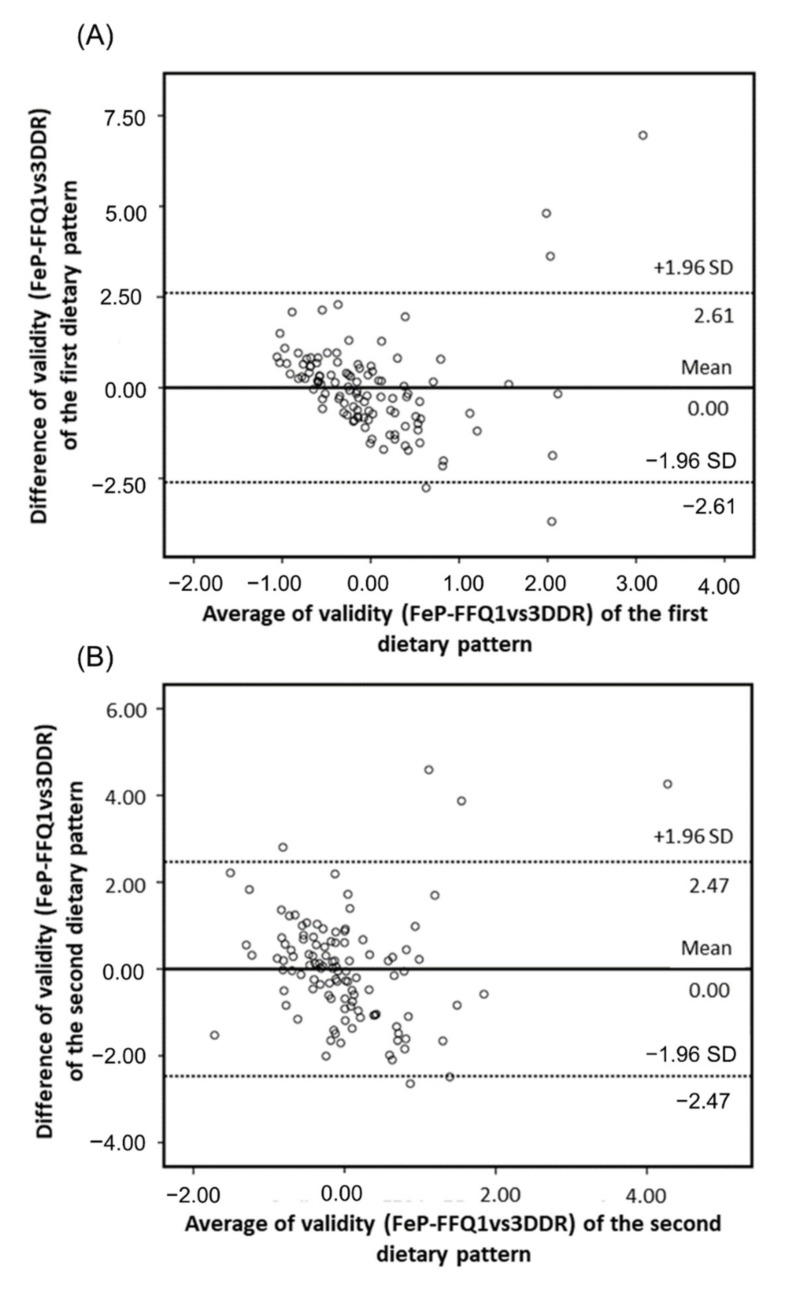
Bland–Altman plots for dietary patterns validity between FeP-FFQ1 and 3DDR: (**A**) “healthy” pattern agreement, (**B**) “industrialized food and dairy” pattern agreement. The solid line represents the mean difference, and the dotted lines represent the agreement limits (SD ±1.96).

Bland–Altman plots compare reproducibility between FeP-FFQ1 and FeP-FFQ2; and validity between FeP-FFQ1 and 3DDR of dietary patterns scores (Figure 1 and Figure 2, respectively). The *x*-axis shows dietary pattern mean score, and the *y*-axis, the difference between scores, FeP-FFQ1 vs. FeP-FFQ2 for reproducibility and FeP-FFQ1 vs. 3DDR for validity.

It is noteworthy that the means for reproducibility and validity of both patterns was equal to zero. This allows identifying the agreement magnitude visually between both methods, in this case, of FeP-FFQ and 3DDR. Dotted lines show the agreement limits between both methods, and they are situated at ±1.96 SD from the mean agreement. The plot shows that most dietary pattern scores fall within the agreement limits.

## 4. Discussion

This study shows the relative reproducibility and validity of a qualitative food frequency questionnaire to identify iron-related dietary patterns (FeP-FFQ) among Mexican pregnant women. This is relevant considering the high prevalence of anemia among Mexican pregnant women (34.9%) [2], which is similar to the global prevalence (36.5%) [1] and because this prevalence doubled from 2012 to 2018, going from 17.9% to 34.9% [2]. Moreover, the anemia prevalence among Mexican pregnant women is significantly higher than in non-pregnant women, highlighting the importance of having a tool that helps to evaluate the iron-related dietary patterns among this population.

### 4.1. Reproducibility and Validity of FeP-FFQ

The FeP-FFQ showed sufficient reproducibility and validity indicators in the food items to identify iron-related dietary patterns in pregnant women. Moreover, this affirmation is supported by the obtained reproducibility coefficients (r > 0.3) for the items most frequently consumed. Other studies have reported correlation coefficients similar to those in this study [15,18,26,27]. Regarding the time interval in the administration of the questionnaires, it should be noted that in the present study, an interval of one month was used between the administration of FeP-FFQ1 and FeP-FFQ2, obtaining reproducibility coefficients greater than those expected. In contrast, the studies by Rodríguez [27] y Glabska [15] used wider intervals (one year and six weeks, respectively) to evaluate questionnaire reproducibility. The obtained coefficients were similar to those in this study (r > 0.3). On the other hand, the variability of the obtained coefficients could be explained by diverse factors: (a) the statistical analyses used: in this study, only the Spearman method with qualitative variables of frequency intake was used, while Baer [28] used the Pearson method quantitatively and adjusted to the total energy intake. (b) Participants characteristics: the present study included women between 20 and 40 years of age, although studies in other countries have included adolescent pregnant women; thus, the differences observed between these populations could be explained by the differences in the diet of adolescents [26,29].

The FeP-FFQ obtained validity correlation coefficients of >0.3 for the most frequently consumed items, similar to the values reported by Robinson [30], Galante [31], and Brunst [32]. However, other studies have reported slightly higher validity coefficients (r > 0.6) [29,33,34]. These differences could be attributed to diverse factors: the design of the questionnaire, including the number of items, seasonality, and time-frame can contribute to the coefficients’ variability between studies; the variability in the methodology used, for example, the reference standard used as a method for validation may also contribute [35,36,37]. The reference standards most commonly used are diet records (DR), 24h-R, and the biochemical markers [17,27,35,36,38]. Generally, 24h-R and DR cannot represent the habitual consumption of the period of interest since they often contain errors and underestimate the intake of nutrients by almost 20% [36]. Therefore, correlation coefficients between both methods for most foods and nutrients are between 0.4 and 0.7 [36]. Nevertheless, DRs are deemed the best comparison method available [19]. The present study used one DR as the reference standard, a method used by different studies that obtained similar validity coefficients [15,18,26,29,30,39,40,41,42].

### 4.2. Reproducibility and Validity of Dietary Patterns

Dietary patterns offer information about the characteristic combinations of the foods consumed by individuals or groups. The methodology to define them is relatively new and has become increasingly popular in nutrition epidemiology to evaluate a diet as an alternative to nutrient-based analyses [43,44,45,46,47,48]. The factorial analysis uses reported information from different instruments to identify common underlying dimensions (factors or patterns), where food intake or several foods are integrated through scores [43,46,47]. Subsequently, these are labeled, but this method can be subjective; sometimes it tends to confuse the population because it is done based on the foods patterns [46].

In the present study, the first dietary pattern was labeled “healthy”, similar to the patterns reported by some other studies [18,45,47,49]. “Industrialized food and dairy” was the label for the second pattern, which was not found in similar studies screened by the authors [18,43,44,45,46,47,48,49]. Few studies have examined the validity and reproducibility of dietary patterns obtained through factorial analysis [48].

In this study, Pearson correlation coefficients for reproducibility of the dietary pattern scores were 0.60 for the “healthy” pattern and 0.55 for the “industrialized food and dairy” pattern. These results are very similar to the ones reported in another three studies analyzing reproducibility of dietary pattern scores [43,45,48]; however, these studies did not analyze iron-related dietary patterns.

Validity correlation coefficients obtained in this study were “good”, with 0.11 for the “healthy” dietary pattern and 0.20 for the “industrialized food and dairy” pattern. Other studies have reported higher dietary pattern scores correlation values for validity [18,43,44,45,46,47,48]. For example, Beck et al. [18] reported reproducibility and validity coefficients higher than those in the present study. These discrepancies could be attributed to the population characteristics. Our study included pregnant women from 20 to 40 years of age, mainly with secondary school studies and a medium-low socioeconomic status, while Beck [18] included women between 18 and 44 years of age, not pregnant, with high education studies and high socioeconomic status. In this regard, Teixeira y col. [50] reported that age, education level, and socioeconomic status were directly associated with Brazilian pregnant women’s dietary patterns; furthermore, they suggested there is a need for additional resources to allow vulnerable mothers to have an adequate diet during pregnancy.

The present research is the first study of its type that complemented its results with ICC for the reproducibility and validity of both patterns. However, the values for validity (“healthy” = 0.36 and “industrialized food and dairy” = 0.37) were not as good as the ones for reproducibility (“healthy” = 0.76 and “industrialized food and dairy” = 0.71). Because correlation coefficients can underestimate the level of agreement with actual intake between the reference method and the new method [35,51], this study additionally used the Bland–Altman analysis to evaluate the level of agreement between methods. The mean difference of the scores for both dietary patterns was equal to zero, similar to the result reported by Beck et al. [18] for their validated dietary patterns.

The “healthy” and “industrialized food and dairy” dietary patterns scores for FeP-FFQs and 3DDR were classified into tertiles. Both patterns were classified for the same third: 58.2% and 51.9%, and opposite third: 5.5% and 11.9%, respectively. Similar results were reported by Beck [18], finding that “healthy” and “sandwich and drinks” patterns classified in the same third: 52.17% and 54.78%; and opposite third: 52.17% and 54.78%, respectively.

In our study, weighted Cohen’s kappa coefficient was used to quantify the relative importance between agreements and different levels of disagreement [52]. The agreement for relative validity was fair and significant for both dietary patterns, in contrast to the moderate agreement for validity found by Beck [15]. On the other hand, both dietary patterns showed a moderate reproducibility, similar to the one reported by Beck [18]. It is noteworthy that the use of weighted Cohen’s kappa is controversial, given that its magnitude depends on the number of categories used and the weights applied, since the maximum weight is given to perfect agreement and proportionally lower weights according to the level of agreement [51,53].

### 4.3. Limitations and Strengths

This study presents some limitations, which are listed next. The use of 3DDR as a reference standard for validity could have been biased if the participants made mistakes in their reported intake; regardless, participants were trained to minimize these potential mistakes. Additionally, it has been reported that people tend to change their habitual intake when they know they are under evaluation [19], which may cause the wrong estimation of the actual intake. Nonetheless, it was decided to use this method because it minimizes potential memory biases of the 24h-R and reduces the time of administration, though they must be screened in detail through interviews. A card with the nine consumption frequencies suggested by Walter Willet [19] was used to minimize errors and reduce time during the FeP-FFQ application. This card was provided to every participant, and they indicated the frequency of intake of each food item.

Another limitation could be that all of the 32 food items most frequently consumed were included in the factorial analysis, which is different from the one used in other studies, which tend to group all consumed foods into categories [45,47,49,54,55]. However, it has been described that consuming 15 to 20 food items per week is possible for most people, even though optimal health can be achieved when a variety of 30 or more food items are consumed per week [56]. Including 32 food items in the analysis allows identifying poor dietary patterns through to the most healthy patterns. In the present study, biochemical markers were not included due to lack of feasibility to obtain serum ferritin and C-reactive protein as primary indicators for iron deficiency. A further study is planned to investigate if the identified dietary patterns are related to iron deficiency biochemical markers.

Lastly, in this study specific nutrients known to affect the absorption of iron, such as phytic acid/phytates, polyphenols/tannins, proteins from soya beans, and calcium [57], were not considered for analysis.

The strengths of this study are as follows: the FeP-FFQ included food items that have been identified as facilitators or inhibitors of iron absorption and the sources of this nutrient, which provides a complete picture of its consumption. In addition, to the best of our knowledge, the FeP-FFQ is the first tool designed specifically to determine iron-related dietary patterns, also reporting the reproducibility and validity of these dietary patterns for its use among pregnant women. Although a previous similar study was identified^15^, this was conducted among women aged 18 to 44 years from New Zealand, and it did not focus on identifying iron-related dietary patterns for pregnant women.

## 5. Conclusions

The FeP-FFQ showed good indicators of reproducibility and validity; therefore, this instrument could contribute to identifying iron-related dietary patterns among pregnant women since it provides an efficient way to evaluate iron intake qualitatively and the influence of other diet components on iron absorption (facilitators and inhibitors). In addition, this instrument could be helpful to determine how the combination of foods and drinks might affect iron status and thus, be able to provide practical clinical and public health information on the prevention of iron deficiency in pregnant Mexican women.

Because the application of the FeP-FFQ is easy, it will allow further studies to analyze the relationships between dietary patterns, iron status, and its risk factors among pregnant women and the baby. Likewise, because this tool is quick to be applied, simple, and relatively inexpensive, it might be helpful in epidemiological studies to determine the iron intake of pregnant women qualitatively, contributing to the early detection of risk factors related to iron deficiency and anemia prevention among Mexican pregnant women, and in consequence, reduction in health expenses.

Moreover, the implementation of this tool could also be helpful to develop and/or evaluate programs and public policies aiming at the optimal iron status of populations and, therefore, healthier pregnancies and babies. Further studies might explore the associations between dietary patterns and iron-deficiency anemia, through biomarkers, for a complete picture of the validity of this tool.

## Figures and Tables

**Table 1:** Participants’ sociodemographic, anthropometric characteristics and iron status (*n* = 110).

Sociodemographic Characteristics		*n* (%)
Civil status	Married	33 (30)
	Free union	60 (54.5)
	Single	16 (14.5)
	Divorced	1 (1)
Education level	None	0 (0)
	Knows how to write and read	1 (0.9)
	Elementary school	12 (10.9)
	Secondary school	51 (46.4)
	High school	36 (37.2)
	Bachelor degree	9 (8.2)
	Graduate degree	1 (0.9)
Occupation	Housewife	89 (80.9)
	Paid job	19 (17.3)
	Student	2 (1.8)
Socioeconomic status *	AB (high class)	3 (2.7)
	C+ (middle-high class)	5 (4.5)
	C (middle class)	19 (17.3)
	C− (middle-low class)	22 (20)
	D+ (low-middle class)	35 (31.8)
	D (low class)	26 (23.6)
	E (extreme poverty)	0 (0)
**Anthropometric characteristics**		
Height (m)	Mean (SD)	1.6 (0.1)
Pregestational weight (kg) **	Mean (SD)	66.1 (15.2)
Pregestational BMI ***	Mean (SD)	26.0 (5.7)
Pregestational BMI status	Low	11 (10)
	Normal	48 (43.6)
	Overweight	25 (22.7)
	Obese	26 (23.6)
Current weight (kg)	Mean (SD)	73.2 (15.2)
Current BMI	Mean (SD)	28.8 (5.6)
Current BMI status ****	Low	10 (9.1)
	Normal	35 (31.8)
	Overweight	39 (35.5)
	Obese	26 (23.6)
Iron status		
Hemoglobin (g/dL) *****	Mean (SD)	12.15 (1.04)
Hemoglobin status *****	Anemia (≤11.0)	16 (14.5)
	Normal (≥11.1)	94 (85.5)

SD: standard deviation; BMI: body mass index. * Obtained and classified using the AMAI (Mexican Association of Market Intelligence and Opinion Agencies) scores [25]. ** Obtained by selfreport at the initial interview. *** Calculated from the selfreported pregestational weight. **** Atalah E, Castillo C, Castro R, Aldea A. Propuesta de un nuevo estándar de evaluación nutricional en embarazadas. Rev Med Chile 1997; 15: 1429–1436 [23]. ***** Hb status from the 3rd trimester of pregnancy.

**Table 2:** Estimated intake and comparisons between FeP-FFQ1, FeP-FFQ2 & 3DDR (*n* = 110).

		Frequency of Intake over 3 Days Mean ± SD		Difference Mean ± SD		Correlation Coefficients ^®^ †	
Items	FeP-FFQ1	FeP-FFQ2	3DDR	FeP-FFQ1 vs. 3DDR	FeP-FFQ1 vs. FeP-FFQ2	FeP-FFQ1 vs. 3DDR	FeP-FFQ1 vs. FeP-FFQ2
Water	15.54 ± 6.07	15.62 ± 5.69	20.30 ± 8.56	−4.84 ± 8.20 **^b^	−0.17 ± 5.73	0.41 **	0.52 **
Corn tortilla	6.07 ± 3.56	5.78 ± 3.49	4.30 ± 1.78	1.77 ± 3.41 **^a^	0.28 ± 3.19	0.33 **	0.59 **
Tomato	4.91 ± 4.96	3.94 ± 4.11	2.78 ± 1.52	2.12 ± 5.17 **^a^	0.96 ± 4.56 *	0.01	0.50 **
Citrus fruits ^c^	4.20 ± 4.57	2.72 ± 3.38	1.92 ± 1.54	2.28 ± 4.80 **^a^	1.48 ± 3.94 **^a^	0.01	0.54 **
Lime ^d^	4.06 ± 5.12	3.05 ± 3.72	1.36 ± 1.37	2.69 ± 4.91 **^a^	1.00 ± 3.42 *	0.28 *	0.74 **
Onion	3.40 ± 3.72	3.16 ± 3.16	2.51 ± 1.65	0.89 ± 3.92 *	0.24 ± 3.84	0.09	0.38 **
Milk	3.32 ± 3.55	3.09 ± 2.63	2.73 ± 1.76	0.59 ± 3.38	0.23 ± 3.20	0.34 **	0.49 **
Stone fruits ^e^	3.08 ± 3.91	2.13 ± 3.27	0.76 ± 0.99	2.31 ± 3.80 **^b^	0.94 ± 3.43 *	0.23 *	0.55 **
Homemade beans	2.64 ± 2.75	2.25 ± 2.17	1.45 ± 1.25	1.19 ± 2.83 **^a^	0.39 ± 2.08 *	0.15	0.68 **
Chilies ^f^	2.64 ± 3.32	2.10 ± 2.47	2.17 ± 1.84	0.46 ± 3.18	0.53 ± 2.92	0.35 **	0.52 **
Banana and fried plantain	2.46 ± 3.57	1.94 ± 2.70	1.19 ± 1.09	1.27 ± 3.74 **^a^	0.52 ± 3.71	−0.00	0.32 **
Apple	2.21 ± 3.62	1.62 ± 3.11	0.65 ± 0.86	1.56 ± 3.59 **^a^	0.59 ± 2.49 *	0.15	0.73 **
Lettuce	1.86 ± 2.35	1.36 ± 2.02	0.76 ± 0.98	1.09 ± 2.51 **^a^	0.49 ± 2.56 *	0.04	0.31 **
Carrot	1.69 ± 2.53	1.21 ± 1.37	0.74 ± 0.99	0.95 ± 2.57 **^a^	0.48 ± 2.91	0.15	0.23 **
Soda	1.62 ± 2.55	1.89 ± 2.75	1.35 ± 1.49	0.27 ± 2.15	−0.27 ± 2.09	0.54 **	0.69 **
Eggs	1.58 ± 2.12	1.36 ± 2.02	1.51 ± 1.06	0.07 ± 2.03	0.22 ± 2.19	0.33 **	0.17 **
Melon	1.53 ± 3.80	0.93 ± 2.28	0.34 ± 0.56	1.19 ± 3.78 **^a^	0.60 ± 2.84 *	0.12	0.66 **
Watermelon	1.41 ± 3.54	0.45 ± 0.90	0.22 ± 0.47	1.18 ± 3.52 **^a^	0.96 ± 3.35 *	0.11	0.33 **
Sour cream	1.39 ± 1.58	1.29 ± 1.49	0.86 ± 0.96	0.52 ± 1.67 **^a^	0.10 ± 1.14	0.21 *	0.72 **
Rice	1.35 ± 1.51	0.94 ± 0.56	0.69 ± 0.87	0.65 ± 1.66 **^a^	0.41 ± 1.47 *	0.10	0.25 *
Yogurt ^g^	1.26 ± 1.28	1.12 ± 1.37	0.43 ± 0.67	0.82 ± 1.23 **^b^	0.14 ± 1.04	0.34 **	0.69
Pasta soup	1.19 ± 1.63	1.11 ± 1.28	0.55 ± 0.73	0.63 ± 1.68 **^a^	0.07 ± 1.68	0.15	0.34 **
Potato	1.15 ± 1.55	1.00 ± 0.96	0.87 ± 0.91	0.28 ± 1.78 **	0.15 ± 1.07	0.02	0.73 **
Mexican sweet bread	1.13 ± 1.83	0.83 ± 0.83	0.75 ± 0.93	0.37 ± 1.98 *	0.29 ± 1.65	0.09	0.43 **
Cookies	1.12 ± 1.99	0.95 ± 1.34	0.56 ± 1.98	0.55 ± 2.82 *	0.16 ± 1.94	0.00	0.37 **
Poultry	1.09 ± 1.62	0.88 ± 0.82	0.81 ± 0.77	0.28 ± 1.77	0.21 ± 1.70	0.04	0.16 **
Cheese	1.07 ± 1.54	0.96 ± 1.55	0.66 ± 0.86	0.40 ± 1.79 *	0.10 ± 0.74	−0.03	0.88 **
Papaya	1.00 ± 2.02	0.82 ± 1.94	0.31 ± 0.63	0.69 ± 2.04 **^a^	0.17 ± 1.94	0.13	0.51 **
Ice-cream, sorbet, popsicles	0.93 ± 1.81	0.67 ± 1.21	0.09 ± 0.31	0.83 ± 1.80 **^a^	0.26 ± 1.53	0.15	0.54 **
Grains	0.91 ± 1.87	0.70 ± 1.15	0.45 ± 0.69	0.46 ± 1.79 *	0.20 ± 1.65	0.30 **	0.48 **
Beef	0.90 ± 0.61	0.82 ± 0.57	1.70 ± 1.28	−0.79 ± 1.32 **^b^	0.08 ± 0.60	0.17	0.47 **
Sausages and ham	0.90 ± 1.32	0.97 ± 1.33	0.95 ± 0.95	−0.58 ± 1.52	−0.07 ± 1.17	0.14	0.61 **

* Significant values (*p* < 0.05); ** Significant values (*p* < 0.01); **^®^** † Spearman correlation coefficients; ^a^ Medium effect size (0.3–0.49); ^b^ Large effect size (≥0.5) [20]. ^c^ Includes orange or mandarin, guava, lemon, pineapple, grapefruit, and strawberries; ^d^ used in flavor water, salads, broths, meat); ^e^ Such as mango and peach; ^f^ Fresh, dried, minced or whole; ^g^ Drinking yogurt included.

**Table 3:** Factor loadings for the two identified dietary patterns by administration of FeP-FFQ1, FeP-FFQ2, and 3DDR (*n* = 110).

	Dietary Pattern					
	Healthy			Industrialized Food and Dairy		
Items	FeP-FFQ1	FeP-FFQ2	3DDR	FeP-FFQ1	FeP-FFQ2	3DDR
Melon	0.84	0.77	0.48			
Watermelon	0.78	0.19	0.29		0.39	−0.24
Banana and fried plantain	0.76	0.34	0.35			−0.42
Citrus fruits	0.75	0.67	0.50		0.39	
Stone fruits	0.75	0.72	0.37		0.23	
Grains	0.63	0.44		0.25		−0.46
Apple	0.62	0.83	0.69			
Potato	0.60			0.23	0.56	
Lime	0.59	0.66	0.56			0.21
Tomato	0.51	0.66		0.39		0.52
Eggs	0.49			0.20		
Onion	0.48	0.53				0.70
Papaya	0.47	0.30	0.30	−0.26	0.31	
Homemade beans	0.42	0.38				
Lettuce	0.39	0.73				0.22
Carrot	0.38	0.71	0.32			
Rice	0.36	0.33		0.35	0.45	0.27
Corn tortilla	0.34	0.24		0.42		0.33
Milk	0.28	0.25		0.48		−0.42
Chilies (fresh, minced, dried, whole)	0.27	0.32	0.44			0.44
Ice cream, sorbet, popsicles	0.22			0.34	0.44	0.29
Beef	0.20		0.39			0.40
Soda			−0.26	0.38	0.54	0.40
Pasta soup				0.77	0.34	0.21
Sausages and ham				0.66	0.61	0.20
Cheese				0.63	0.39	−0.30
Sour cream			0.25	0.44	0.52	
Mexican sweet bread			−0.22	0.21	0.48	
Cookies			0.40	0.15	0.41	
Yogurt			0.33	0.42	0.40	
Poultry				0.60	0.31	
Water			0.25	0.32		

Notes: Correlations < 0.15 excluded to enable ease in interpretation.

## Data Availability

Not applicable.
